# Aerosol delivery during invasive mechanical ventilation: development of a preclinical *ex vivo* respiratory model for aerosol regional deposition

**DOI:** 10.1038/s41598-019-54480-9

**Published:** 2019-11-29

**Authors:** Yoann Montigaud, Quentin Georges, Jérémie Pourchez, Lara Leclerc, Clémence Goy, Anthony Clotagatide, Nathalie Prevot, Sophie Perinel-Ragey

**Affiliations:** 1 Mines Saint-Etienne, Univ Lyon, Univ Jean Monnet, INSERM, U 1059 Sainbiose, Centre CIS, F - 42023 Saint-Etienne, France; 2 CHU Saint-Etienne, Saint-Etienne, F-42055 France; 3 INSERM U1059 Sainbiose, Université Jean Monnet, Saint-Etienne, F-42023 France

**Keywords:** Translational research, Preclinical research

## Abstract

In intensive care units, nebulization is a usual route for drug administration to patients under mechanical ventilation (MV). The effectiveness of inhalation devices as well as depositions sites of aerosols for ventilated patients remain poorly documented. *In vivo* human inhalation studies are scarce due to ethical restrictions because imaging techniques require radioaerosols to assess regional aerosol deposition. Thus, we developed an *ex vivo* respiratory model under invasive MV for preclinical aerosol deposition studies. The model was composed of *ex vivo* porcine respiratory tracts. MV was achieved thanks to a tracheal intubation and a medical ventilator under controlled conditions. Respiratory features were studied using analogical sensors. Then regional homogeneity of gas-ventilation was assessed with ^81m^Krypton scintigraphies. Finally, a proof of concept study for aerosol deposition was performed. Obtained respiratory features as well as gamma-imaging techniques, which demonstrated a homogenous regional ventilation and about 18% ± 4% of the nebulized dose deposited the respiratory tract, were in good agreement with human data available in the literature. This original *ex vivo* respiratory model provides a feasible, reproducible and cost-effective preclinical tool to achieve aerosol deposition studies under MV.

## Introduction

In intensive care units (ICU), nebulization is a usual route to deliver drugs in the respiratory tract of patients under mechanical ventilation (MV). Aerosol therapy is used by up to 99% of intensivists according to a recent international survey^1^. Inhaled drug administration to mechanically ventilated patients is an attractive pathway for a large amount of therapeutics (such as bronchodilator treatments, antibiotics and systemic medications). However, therapeutic index of many aerosolized treatments, defined as the quantitative comparison of doses causing toxicity and therapeutic effect, is largely variable^1^. Inhaled therapeutics often lack of robust scientific evidence proving their clinical efficacy^2^. As an example, the guidelines of the European Respiratory Society^3^ underlined that even if metered-dose inhalers (MDI) and nebulizers are usually used in ICU to deliver bronchodilators to mechanically ventilated patients^2^, it is not yet known which modality of treatment is more effective. This is related to the complexity to perform studies with an important level of evidence and allowing the measurement of meaningful outcomes such as morbidity, mortality and duration of mechanical ventilation^2^. Moreover, for ICU conditions - *e.g*. for patients suffering of acute respiratory failure (ARF) - the accurate dose and anatomic targets of inhalation therapy are quite poorly documented. Indeed, ICU patients frequently suffers of ARF: a prevalence study in 81 ICU claimed that ARF represented 24% of main admission diagnosis and aerosolized drug administration was frequently considered in this situation^4^ (49% of these patients received inhaled therapies). According to severity and etiology, it is an usual indication for invasive MV^5^. This MV, requiring nowadays a positive airway pressure ventilation is very different from physiological ventilation (negative pressure ventilation thanks to the pleural cavities). Consequently, nebulization efficiency and deposition pattern within the respiratory tract is largely impacted by MV^6^.

Regarding the variety of therapeutic agents and diseases, the implementation of optimal nebulization conditions for patients under MV are ongoing challenges in ICU. In addition to the variety of medicines, pharmacokinetics and therapeutic index, many factors have been shown to affect the efficiency of nebulized treatments by modifying the aerosol regional deposition for mechanically ventilated patients^6^: angulation of tracheal intubation^7^, artificial airways^7^, respiratory pattern^8^, design of nebulizers^9^, presence of heated humidification^10^, position of the nebulization device in the ventilation circuit^11^, etc. For example, clinical trials studying bacterial eradication following administration of nebulized antibiotics struggled to demonstrate a significant difference compared to intravenous therapeutics only^12,13^. Moreover, some drugs such as bronchodilators showed a wide therapeutic index and thus, no influence of the different conditions of nebulization (type of nebulizer, position in the ventilator circuit, humidification condition and ventilation mode) could be observed in clinical studies^1^.

A recent systematic review^14^ aimed to assess inhaled drug delivery on mechanically ventilated patients and existing animal models. This review supports the conclusion that lung deposition of nebulized treatments in mechanically ventilated patients is lower than 20% of nominal dose delivered by nebulizers and mostly occurs in proximal airways. However, a limited number of *in vivo* studies assess lung deposition of inhaled drugs during MV. As a matter of fact, quantitative nuclear medicine imaging is the gold standard to assess regional aerosol deposition and consequently *in vivo* human inhalation studies are scarce due to ethical restrictions related to radioactivity and arduousness of nuclear medicine procedures on MV patients. Besides, results generated using *in vitro* experiments or various animal models remain controversial due to wide differences between these models and human airways in size and bronchial divisions but, also, in ventilation physiology^15,16^.

To sum up, in patients receiving MV, it is a challenge to ensure that nebulizers deliver aerosol to distal airways because currently, the proportion of the drug that effectively reaches the lung is always quite low. Thus, nebulization devices and administration procedures have to be improved for patients under MV. All these considerations explain the need to achieve preclinical aerosol studies using a robust respiratory model, practicable, cost-effective, without restrictive ethical issues, well adapted to MV and showing a suitable comparability to human anatomy and physiology. These experimental preclinical studies would aim to assess the impact of several parameters on the deposited lung dose as well as regional aerosol deposition. First, the performance of aerosol devices has to be studied: aerosol size distribution and aerosol output depending of the technology (vibrating mesh or jet nebulizers, MDI etc.) Then, the different components of aerosol administration procedure: continuous *vs*. breath-synchronized mode, the presence of heating humidifier, the nebulizer position in the ventilator circuit. This multiplicity of parameters implies the necessity of modeling.

Our research group previously developed a preclinical respiratory model to assess the aerosol deposition in spontaneous ventilation^17,18^. Thus, based on this original know-how, this work aimed to develop and then validate a reproducible and relevant *ex vivo* respiratory model dedicated to preclinical aerosol studies under MV. At first, a physiological study was conducted using a pneumotachograph and a differential pressure sensor to determine whether the developed respiratory model satisfactorily fitted human ventilation data available in literature^19,20^. Then, ^81m^Krypton (^81m^Kr) scintigraphic study was performed by gamma-imaging in order to assess the homogeneity of regional gas-ventilation. As a proof of concept, the relevance of the developed model for preclinical inhalation studies was assessed by the quantitative imaging of regional aerosol deposition using a radiolabeled aerosol (^99*m*^ technetium-labeled diethylene-triamine-pentaacetic acid, ^99m^Tc DTPA) generated by a mesh nebulizer.

## Results

### Anatomical features

Overall, 37 anatomical models were set up for the whole study:

- (i) Concerning the 21 respiratory tracts used for the physiological study, the weight ranged from 659 to 1099 g (mean ± standard deviation [SD]: 852 ± 112 g,). A median of 2 cuts (range 0–4) and a median of 6 sutures (range 0–22) per respiratory tract were performed.
- (ii) Concerning the 10 respiratory tracts used for ^81m^Kr ventilation scintigraphies, the weight ranged from 741 to 930 g (mean ± SD: 853 ± 74 g). A median of 2 cuts (range 0–4) and a median of 1 suture (range 0–8) per respiratory tract were performed.
- (iii) Concerning the 6 respiratory tracts used for ^99m^Tc DTPA scintigraphies, the weight of the IT tract ranged from 749 to 1165 g (mean ± SD: 875 ± 216 g). A median of 1 cuts (range 0–2) and a median of 0 sutures (range 0–6) per respiratory tract were performed.

Concerning the mass of the respiratory tracts, distribution for each type of experiment followed Gaussian distribution. One-way ANOVA showed no significant difference. Concerning the number of wounds of the respiratory tracts, distribution for each type of experiment did not follow Gaussian distribution. Kruskall-Wallis test showed no significant difference. Concerning the number of sutures of the respiratory tracts, distribution for each type of experiment did not follow Gaussian distribution. Kruskall-Wallis test showed a significant difference (approximate *p* = 0,0146).

### Physiological features

Physiological measurements were performed using 21 different *ex vivo* porcine respiratory tracts which were connected to the same ventilation circuit and ventilator. On this series of 21 lung models, no significant correlation between the leaks and the number of cuts or the number of suture could be found, with correlation coefficient of 0.2081 and 0.4056, respectively. Physiological values obtained from pneumotachograph and differential pressure sensor over a 3 minutes ventilation (59 respiratory cycles) for the 21 respiratory tracts are shown in the table below (Table 1).

**Table 1:** Physiological data for the 21 porcine respiratory tracts over a 3 minutes controlled mechanical ventilation. SD: standard deviation.

	Tidal volume (mL)	Minute ventilation (L/min)	Leaks (%)	Resistance (cmH_2_O.L^−1^.sec^−1^)	Compliance (mL.cmH_2_O^−1^)
Mean	365	7.26	15.74	6	30
SD	30	0.60	7.02	1	3
Range	[282; 417]	[5.64; 8.33]	[3.63; 33.70]	[5; 8]	[22; 34]

### ^81m^Kr ventilation scintigraphies

**Figure 1:**
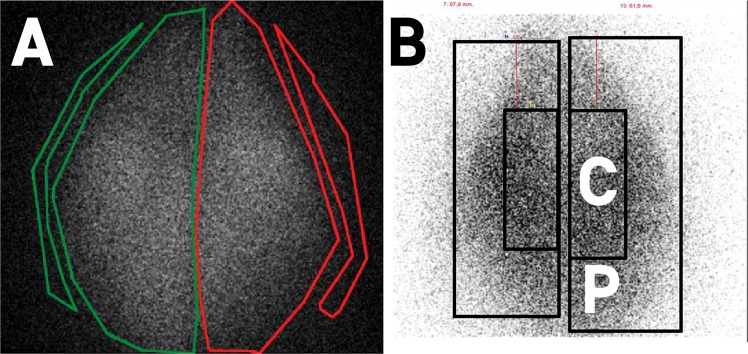
^81m^Krypton planar scintigraphic images showing homogenous ventilation of porcine respiratory tracts. (**A**) ROIs of each lung and respective background are materialized in green for the right lung and in red for the left lung; (**B**) peripheral (P) and central (C) ROIs for each lung.

The ^81m^Kr ventilation scintigraphies (Fig. 1A,B) were performed on 10 different *ex vivo* models. Different regions of interests (ROIs) were used for calculation and are visible on Fig. 1. The Fig. 1A displays left and right lungs with 2 background noises ROI; the Fig. 1B displays central and peripheral lung regions. Ratios of the count rates between peripheral to central regions and of left lung to total count were calculated to compare with human study using these original markers (Table 2).

**Table 2:** Relative uptake measurements based on ^81m^Krypton planar scintigraphies and literature comparisons.

	Left/total ratio	Right/total ratio	Right/Left ratio	Right/left ratio from Dugernier *et al*.^8^	PI
Mean	48%	52%	1.11	1.09	52%
SD	4%	4%	0.18	0.32	4%
Range	[39%; 52%]	[49%; 61%]	[0.91; 1.56]	NA	[45%; 59%]

SD: standard deviation; PI: penetration index; NA: data non available.

First, the ratio of the left lung-to-total count was 48 ± 4%. The ratio of the right lung-to-total count was 52 ± 4%. Between right and left lungs, no significant differences were observed in terms of ratio to total count. Second, the penetration index (PI), defined as the ratio of the count between peripheral and central regions, was calculated for each lung and showed values of 52±. Data are presented in Table 2.

### ^99m^Tc-DTPA radioaerosol regional deposition

**Figure 2:**
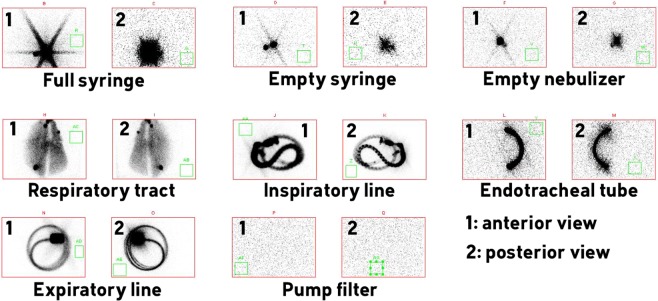
Scintigraphic images of ^99m^Tc-DTPA aerosol deposition obtained after one experiment. The green square in each field of view corresponds to the region of interest used to determine the corresponding background noise.

The ROIs determined after planar scintigraphies allowed to precisely quantify the deposited fractions in each part of the set-up after the nebulization of the radioactive aerosol (Fig. 2 & Table 3). Results in Table 3 are adjusted to 100%.

**Table 3:** Deposition fractions in the different parts of the ventilation circuit and in the model.

		Inspiratory	Expiratory	ETT	RT	Total
Experimental data on the *ex vivo* model	Mean	45%	34%	4%	18%	100%
	SD	10%	9%	2%	4%	N/A
	Range	[30; 64]	[21; 45]	[2; 7]	[12; 23]	N/A
*In vitro* data from Ari *et al*.	Mean		—		24.2%	—
	SD		—		1.2%	N/A
*In vivo* data from Dugernier *et al*.	Mean		84.9%		15.1%	100%
	SD		5%		5%	N/A

Results expressed as fractions of the nebulized dose (relative percentage of the nominal dose that was effectively nebulized). Inspiratory: Inspiratory line. Expiratory: expiratory line + expiratory filter + sealed enclosure filter. ETT: endotracheal tube. RT: respiratory tract or filter at the end of ETT. SD: standard deviation. N/A: not applicable. —: data not available.

Nebulized dose, *i.e*. the mean percentage of the nominal dose that was effectively emitted during the nebulization was 94 ± 7%. Most part of the nebulized dose was deposited in the ventilation circuit but especially in the inspiratory line, which collected 45 ± 10% of the nebulized dose. Expiratory line, expiratory filter and sealed enclosure filter deposited fractions represented 34 ± 9% of nebulized dose. Lastly, respiratory tract fraction accumulated 18 ± 4% of nebulized dose. Experimental data and data obtained from literature are presented in Table 3.

**Figure 3:**
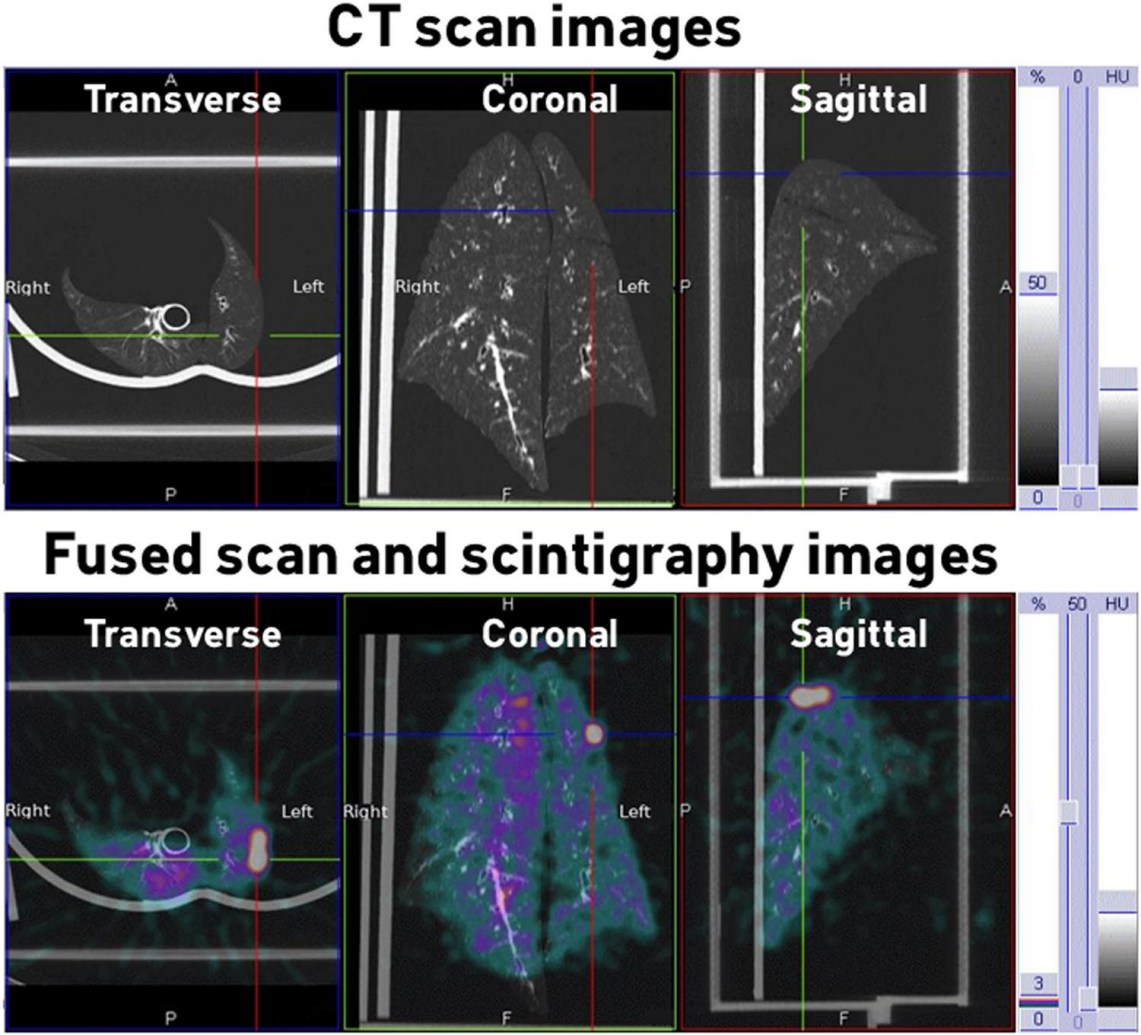
SPECT/CT imaging of one respiratory tract after ^99m^Tc-DTPA nebulization. Upper panels: tomography images. Lower panels: fusion of tomography and scintigraphic images.

SPECT/CT imaging of the lung (Fig. 3) visually showed good penetration as well as a homogeneous anterior and posterior distribution of the aerosol in the lungs.

## Discussion

This work demonstrated that the previously developed *ex vivo* porcine respiratory model for spontaneous ventilation could be successfully adapted for invasive MV. Our results showed a good coherence with human respiratory physiological data of an ICU patient in terms of V_T_, compliance and resistances. Moreover, homogeneity of ventilation, as assessed by ^81m^Kr scintigraphy, was comparable to previous studies ran in spontaneous and mechanical ventilation on human population^8^. Finally, the assessment of radioaerosol regional deposition showed that the extra-pulmonary (mainly the ventilation circuit and the expiratory filter) and lung deposited fractions were in good agreement with previous *in vitro and in vivo* studies^8,10^. Consequently, the developed *ex vivo* respiratory model appeared relevant to perform preclinical studies devoted to aerosol administration for ICU patients under MV.

The anatomical qualities of the porcine respiratory tracts used for the study were fulfilling, as shown by macroscopic qualitative data collected during the dissections, considering that cuts during slaughter were unavoidable. However, despite meticulous suturing, we observed the existence of leaks during physiological measurements and found some extent on the scintigraphies. Leakages were emphasized in positive airway pressure with high levels of positive end expiratory pressure during MV and were assessed as 15.74% ± 7.02 (ratio of inspiratory tidal volume to expiratory tidal volume).

Considering most recent guidelines^21^, the choice of V_T_ of a 8 mL/kg of ideal body weight and other respiratory parameters could be questionable but were based on the recent LUNGSAFE study^22^, which provided detailed information about clinical practices in the management of acute respiratory distress syndrome, a very severe respiratory condition, among ICUs over the world.

Physiological measurements showed good coherence with human data^23^. Respiratory tract resistances remained under 10 cmH_2_O.L^−1^.sec^−1^ as expected with a 7 mm inner diameter endotracheal tube and an inspiratory flow of 35 L/min^24^. Respiratory tract compliance was also consistent with human physiological data of patients suffering ARF^24^. Indeed, the compliance was far from a healthy adult given that the *ex vivo* model is not able to reproduce the human thoracic cage and its mechanical properties. To be precise, the compliance of the model was also modified by the lack of lung perfusion, the *post mortem* lesions to the parenchyma and by the unknown status and quantity of surfactant present into the lungs. Indeed, with a very short half-life, it could be assumed that the surfactant was not functional nor present 24 hours after slaughter. As a result, the proposed respiratory model, with a reduced compliance and under controlled MV, is closed to ARF conditions, explaining the choice of a protective ventilation used for these patients. However, as inhaled drugs are administered to disparate patient populations under various conditions, further studies under assisted ventilation, with different respiratory features or with non-invasive ventilation have to be complementary performed to investigate several nebulization uses.

Despite potential slaughtering concerns (retention of bronchial secretions, minor atelectasis), homogeneity of the ventilation in the right and left lungs depicted by the ^81m^Kr ventilation scans was clearly shown by planar scintigraphies and right/left ratio and PI were close to the repartition in healthy human^8,25^. However, the left/right asymmetry observed both in spontaneous ventilation^25^ and under MV^8^ in humans, wasn’t significatively found on this model. The central position of the middle lobe in our model (due to the absence of rib cage) could be an explanation to this difference.

A large amount of nebulized dose was found both in inspiratory and expiratory line. As recommended by the manufacturer and also confirmed by independent studies^26^, nebulizer was placed as far as possible from the Y piece. The rational is to decrease deposition lost over expiratory line during inspiration. On the other hand it could have led to a larger quantity of aerosol deposition over inspiratory line. Further studies have to be performed with different nebulizer positions on the circuit.

In literature, lung deposition of aerosol generated by vibrating-mesh nebulizers in mechanically ventilated patients was lower than 20% of nominal dose and showed a regional deposition focusing mainly in proximal airways^14^. Values obtained in our study were quite similar, from 12% to 22% (mean ± SD: 18 ± 4%). These results also showed the good concordance of the *ex vivo* respiratory model compared to a previous *in vivo* study^8^ using the same nebulization device and similar administration procedure and ventilation parameters (respiratory tract deposition of 15.1 ± 5%). Similar results were equally available in an *in vitro* study^10^ calculating a respirable dose delivered *via* endotracheal tube, with similar ventilation procedure (filter mimicking lung deposition, mean ± SD: 24 ± 1.2%).

Moreover, this study demonstrated an important nebulized dose compared to the dose initially introduced into the nebulizer tank. Indeed, the Aerogen^®^ Solo vibrating mesh device showed in our work a nebulized dose of 94% (SD 7%) in perfect accordance with literature data reporting a nebulized dose of 96.7 ± 0.7% of the nominal dose^10^. However, one nebulization experiment showed an issue during the nebulization leading to an early stop of the device inducing the presence of a quantitatively important residual volume in the tank at the end of the procedure. Later readings^27^ confirmed that this nebulizer can be randomly interrupted with various retained volumes.

Regarding the humidification of the inhaled gases, no moisture exchange filter was placed between the Y-piece and the endotracheal tube. Indeed, the aerosol particles were likely to obstruct it and it is not recommended to use them during the nebulization^28,29^. Moreover, the use of a heated humidifier would change the particle size distribution of the airborne droplets. Indeed, water contained in the aerosolized particles equilibrates with the surrounding humidity, thus increasing the volume of particles in a water-saturated environment. This has the effect of increasing the particle impaction at the level of the upper airways and thereby decreasing aerosol delivery into the lungs^26,30^. Consequently, despite the possibility allowed by manufacturer, no heated humidifier was used and no gas humidification was performed during this study. This is debatable for several reasons. The punctual interruption of humidification during the nebulization time *in vivo* does not often leave enough time to obtain drought conditions comparable to the *in vitro* manipulations^31^. Nebulization of certain drugs (particularly antibiotics) continuously over long periods does not leave the possibility of a prolonged interruption of humidification. As a result, our findings are probably overstated in relation to clinical practice where the decrease in humidification does not seem to be achievable.

In addition to being physiologically relevant, this new *ex vivo* invasive ventilation model showed several strengths. As porcine respiratory tracts were obtained from porcine slaughterhouses, this model complied with the requirements of the 3 R (replace, reduce, refine) guidelines^32^ for more ethical use of animals in testing. One did not have to sacrifice more animals for the need of these experiments. By their nature, these animal wastes were also cost-effective. When considering the overall models ventilated during this study, data showed a good inter-individual reproducibility with a V_T_ standard deviation of 30 mL. Yet, this model had some limitations. The most important one was the existence of leaks arising directly from the lungs. However, theses leaks did not have any form of impact on ventilation homogeneity as assessed by the ^81m^Kr scintigraphy and some technical improvements are under consideration to reach a better management of these leaks.

This model opens the road to many further preclinical studies running aerosol therapies under various conditions for mechanically ventilated patients. Consequently, it could be the first step to improve efficiency and to power-up the development of aerosol therapy devices for ICU use.

## Material and Methods

### Respiratory tracts

The porcine respiratory tracts were obtained at the release from slaughterhouse, satisfying all the sanitary controls in accordance with French sanitary regulations. All the respiratory tracts were used within 24 hours for sanitary reasons. Otherwise, they were frozen and slowly thawed at ambient temperature 12 hours before the experiment. If any cut was observed on lungs, stitches were realized and data were collected as potentially leading to abnormal leaks with positive pressure ventilation. A bronchoscopy was systematically performed to ensure the absence of significant airway obstruction and all the observations were recorded (obstructions, secretions, need for instillation, bronchial divisions etc.). For each experiment, the porcine respiratory tract was placed in an instrumented sealed enclosure. The tracheal length was standardized to at least 15 cm in order to avoid selective intubation.

### Model set-up and ventilation parameters

The porcine respiratory tracts were mechanically ventilated by means of a tracheal intubation and an ICU Evita 4 ventilator (Dräger, Germany). A self-test procedure followed by a device check was performed before each experiment. The intra-thoracic part was connected to the ventilator using a tracheal tube (Smiths Medical, Portex® SACETT™ Suction Above Cuff Endotracheal Tube, 7.5 mm inner diameter). Tracheal tube was set respecting physiological angulation of 90 degrees (based on mechanically ventilated patients’ tomodensitometry analysis). The ventilation mode was controlled volumetric. Currently, this technique is still mainly used for invasive ventilation in ICUs over the world as it is observed in an international study^33^. Therefore, the ventilation parameters were chosen based on the results of a recent epidemiological study of clinical practice for patients under MV^22^. In this study, patients were ventilated with 8 mL/kg of ideal body weight. As reported by an anthropometric survey published in 2015 by French National Institute of Statistics and Economical Studies, French average height is 175 cm tall leading to a 540 mL tidal volume (V_T_). Positive end expiratory pressure (PEEP) was set to 9 cmH_2_O, inspiratory/expiratory ratio (I/E) to 1/2, and respiratory rate to 20 cycles per minute leading to a predicted minute ventilation of 11 L/min.

As a recent review about aerosoltherapy under MV^26^ recommend it, inspiratory flow was set as low as possible for chosen I/E ratio and respiratory rate leading to 35 L/min.

### Physiological measurements

**Figure 4:**
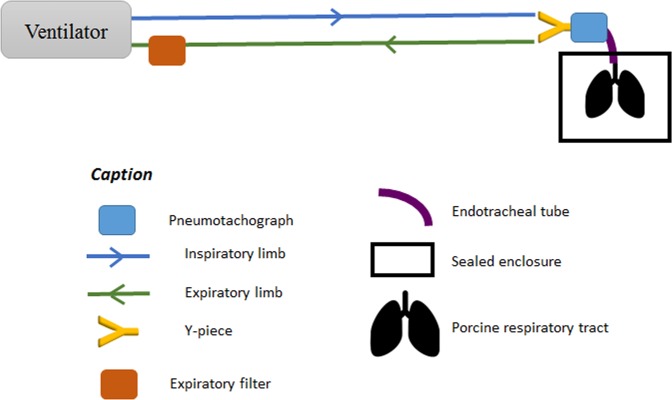
Experiment setup during physiological acquisitions

The first part of the study consisted in recording respiratory physiological features using a Biopac^®^ system (Biopac, Goleta, USA) composed of a pneumotachograph (Pneumotach, TSD 117, Biopac, Goleta, USA) and a differential pressure sensor (TSD160D, Biopac, Goleta, USA). The real-time airflow was recorded between the Y-piece and the tracheal tube using the pneumotachograph (Fig. 4).

These data allowed calculation of the air input and output in the tracheal tube representing the measured minute ventilation. The V_T_ has been obtained by integration of outputs for each respiratory cycle. A median of V_T_ and minute ventilation was then calculated. The differential pressure sensor allowed to calculate median respiratory tract resistance and compliance by following depressions in the enclosure. All data were processed through AcqKnowledge^®^ 5.0 software (Biopac, Goleta, USA).

### 81mKrypton ventilation scintigraphy

The second part of the study consisted in performing ^81m^Kr nuclear imaging to assess the homogeneity of ventilation. The ^81m^Kr ventilation scintigraphies were performed on 10 *ex vivo* models. Due to the short half-life of this radioactive gas (13 seconds) ^81m^Kr ventilation are considered representative of air ventilation^34^. Right and left ratio of total relative uptake corrected for background signal attenuation were calculated for each respiratory tract. Two different regions of interest (ROIs) were identified on ventilation scintigraphies within each lung following Newman *et al*.^35^ recommended method to divide lungs into central and peripheral zones. A central ROI with dimensions equal to half of the width of a whole lung region and one-half of its height was positioned on the interior boundary of the lung, centered by height. The central and peripheral regions represented respectively 25% and 75% of the whole lung region. This allowed to calculate the relative uptake of central and peripheral regions for both left and right lung.

### Aerosol regional deposition using scintigraphy and radiolabeled ^99m^Tc-DTPA aerosol

The third part of the study consisted in a proof of concept for preclinical radioaerosol studies under MV over 6 *ex vivo* models.

The respiratory tract was set to be mechanically ventilated in a 30-degree proclive position, to mimic the widely used position of patients in ICU.

**Figure 5:**
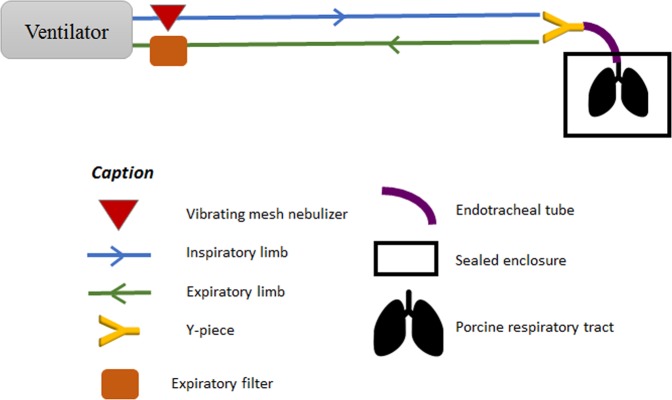
Experimental setup during ^99m^Tc-DTPA nebulization and place of nebulizer on the inspiratory valve

Radioactive aerosols were generated continuously by a vibrating mesh nebulizer (Aerogen Solo^®^, Aerogen Ltd, Galway, Ireland) until the cessation of the nebulizer. The nebulizer was filled with ^99m^technetium-labeled diethylene-triaminepentaacetic acid (^99m^Tc-DTPA, 100 MBq/3 mL). Among all nebulization devices, vibrating mesh are those leading to the best percentage of nominal dose nebulized^6,9^. This type of nebulizing devices usually showed a Mass Median Aerodynamic Diameter (MMAD) ranging from 1 to 5 µm as well as a calculated respirable dose delivered *via* endotracheal tube ranging from 13% to 17%^10^. As recommended by manufacturer to avoid aerosol loss during expiration cycle, the vibrating mesh nebulizer should be placed upstream of the Y-piece. Thus, the nebulizer was placed between the inspiratory inlet and the inspiratory line (Fig. 5) making use of this later one as an aerosol tank as explained by Erhmann *et al*.^26^ when performing continuous nebulization.

Since the aerosol particles generated may damage the flow sensor, it is recommended to place a filter before the expiratory valve^26^. This condition was respected for this study.

Duration of nebulization was set to 15 min based on preliminary test run, in good accordance with previous study from Dugernier *et al*.^8^ showing a 9 minutes nebulization.

### Acquisition of planar gamma-camera images and analysis procedure

The planar scintigraphic images (matrix 256*256) were recorded with a variable angle dual detector SPECT/CT (SYMBIA T2; Siemens, Knoxville, USA) equipped with a low-energy, high-resolution collimator (FWHM 8.3 mm at 10 cm); tested weekly for uniformity (UFOV 533 mm × 387 mm, CFOV 400 mm × 290 mm). Before conducting the inhalation experiments, the initial radioactive dose filled in the nebulizer was quantified (scintigraphic images, 60-sec anterior/posterior, were acquired corresponding to the full and empty syringe). Once the inhalation experiments were performed, 180-sec anterior/posterior images of the experimental setup were acquired for each element: empty nebulizer, inspiratory line, endotracheal tube, Y-piece associated to expiratory line and lungs.

ROIs were delimited on the images with a correction of the background. Calculations took into account the background radiation, physical decay of radioactivity and tissue attenuation correction factors. Consequently to these corrections, as stated by Newman *et al*.^35–37^, some limitations of data quantification in planar imaging are inherent to its 2D nature. It is accepted that errors of no more than 10% in the quantification of whole-lung deposition are tolerated and that mass balance close to 100% validate the method used for attenuation and scatter correction. Hence, the results were expressed as a percentage of activity in relation to the total dose and the nebulized dose, adjusted to 100%. Nominal dose standed for the total amount of radioactivity introduced into the nebulizer.

### Acquisition of 3D gamma-camera images and analysis procedure

A tomography was performed for one respiratory tract. After acquiring 2D images with the same gamma camera (SYMBIA T2; Siemens, Knoxville, USA), SPECT and CT acquisitions were performed immediately with a 2-slice spiral CT for a more rapid and accurate attenuation correction and anatomical mapping. 3D SPECT acquisition of the respiratory tract was performed with 64 (2 × 32) projection images, each of 30 s. Finally, the CT was performed with the following parameters: 130 kV, 90 mAs, 1.25 mm slice thickness, 0.9 mm increment, 1.6 mm pitch, and rotation time of 1.5 s. A multimodality computer platform (Symbia net, Siemens, Germany) was used for image reviews and manipulations. Both the transmission and emission scans were reconstructed using 3D OSEM by default (8 subsets, 5 iterations), with pre-reconstruction smoothing using a 3D Butterworth filter (cutoff: 0.45 cycles/cm; order 5), a 128 × 128 image matrix, a 1.23 zoom, and a pixel size of 3.9 mm. SPECT images were reconstructed using scatter correction (scatter energy window) and CT attenuation correction. CT and SPECT images were matched and fused into trans-axial images. Images obtained were visually compared to 2D imaging, assessing the homogeneous distribution of the aerosol over pulmonary parenchyma.

### Statistical analysis

Results are reported as numbers (%), means with SD. The physiological data were analysed with AcqKnowledge^®^ 5 software (BIOPAC Systems, Inc., of Goleta, California, USA). Statistical analyses were performed using GraphPad Prism^®^ v8.0.2. Briefly, for each type of experiment (*i.e*. physiological study, ^81m^Kr ventilation and ^99m^Tc-DTPA) mass, number of wounds and number of sutures of each respiratory were computed to obtain descriptive statistics. Normality of the distribution was assessed through Shapiro-Wilk test. If Gaussian distribution was shown, one-way ANOVA with Tukey’s *post-hoc* test was used to compare each experiment. If Gaussian distribution was not shown, Kruskall-Wallis’ test with Dunn’s *post-hoc* test to compare each experiment. To study the potential correlation between leaks and wounds and between leaks and sutures, normality was assessed with Shapiro-Wilk normality test, then Spearman correlation tests were performed to determine the significance of the association of each couple of values (*i.e*. leaks-wounds and leaks-sutures).
